# The Neuromuscular Junction: Roles in Aging and Neuromuscular Disease

**DOI:** 10.3390/ijms22158058

**Published:** 2021-07-28

**Authors:** Shama R. Iyer, Sameer B. Shah, Richard M. Lovering

**Affiliations:** 1 Department of Orthopaedics, University of Maryland School of Medicine, AHB, Room 540, 100 Penn St., Baltimore, MD 21201, USA; siyer@som.umaryland.edu; 2 Departments of Orthopaedic Surgery and Bioengineering, University of California San Diego, La Jolla, CA 92093, USA; sbshah@health.ucsd.edu

**Keywords:** NMJ, muscular dystrophy, sarcopenia, myasthenia gravis, exercise

## Abstract

The neuromuscular junction (NMJ) is a specialized synapse that bridges the motor neuron and the skeletal muscle fiber and is crucial for conversion of electrical impulses originating in the motor neuron to action potentials in the muscle fiber. The consideration of contributing factors to skeletal muscle injury, muscular dystrophy and sarcopenia cannot be restricted only to processes intrinsic to the muscle, as data show that these conditions incur denervation-like findings, such as fragmented NMJ morphology and corresponding functional changes in neuromuscular transmission. Primary defects in the NMJ also influence functional loss in motor neuron disease, congenital myasthenic syndromes and myasthenia gravis, resulting in skeletal muscle weakness and heightened fatigue. Such findings underscore the role that the NMJ plays in neuromuscular performance. Regardless of cause or effect, functional denervation is now an accepted consequence of sarcopenia and muscle disease. In this short review, we provide an overview of the pathologic etiology, symptoms, and therapeutic strategies related to the NMJ. In particular, we examine the role of the NMJ as a disease modifier and a potential therapeutic target in neuromuscular injury and disease.

## 1. Introduction

**Figure 1:**
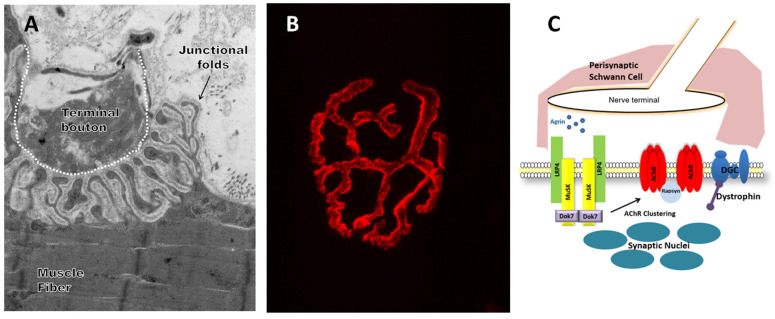
The neuromuscular junction (NMJ) in skeletal muscle. (**A**) Representative electron microscopy image showing ultrastructure of the NMJ. (**B**) Representative confocal microscopy image of fluorescent stain with an acetylcholine receptor binding neurotoxin (α-Bungarotoxin, BTX, red). (**C**) Illustration describing the components of the NMJ. Perisynaptic Schwann cells are glial regulators of NMJ structure and function. Agrin interacts with lipoprotein receptor-related protein 4 (Lrp4), which activates muscle-specific kinase (MuSK). MuSK, a transmembrane tyrosine kinase, through signaling of Dok-7 and rapsyn, drives acetylcholine receptor (AChR) clustering. Dystrophin and its associated glycoprotein complex (DGC), which is present throughout the muscle fiber membrane, accumulate underneath the post-synaptic membrane.

The area of synaptic contact between motor neurons and their target muscle fibers is the neuromuscular junction (NMJ). This synapse occurs at a specialized area of the sarcolemma called the endplate (Figure 1). The “pretzel-shape” of a typical, healthy endplate in mammalian muscle results from the local arborization of the motor neuron at its terminal. The distal aspect of each branch of the motor neuron is enlarged and forms pre-synaptic boutons, which contain synaptic vesicles filled with the neurotransmitter acetylcholine (ACh). Boutons directly overlie post-synaptic invaginations of the sarcolemma called junctional folds [1], upon which high-density clusters of acetylcholine receptors (AChRs) reside. Interdigitated between post-junctional folds are the perisynaptic Schwann cells (PSCs), which are glial regulators of NMJ structure and function [2]. When released into the synaptic cleft, ACh binds to AChRs, causing an endplate potential (EPP), a local depolarization that then propagates throughout the muscle fiber as a conducted action potential.

Accumulating evidence has made it clear that the NMJ in mature skeletal muscle is not a fixed permanent structure [3,4] but instead is continually remodeling, thereby possessing a large degree of functional plasticity [5]. Most dramatically, the NMJ becomes fragmented and ultimately dissolves following nerve transection (denervation), concurrent with axon withdrawal [6,7]. PSC ablation also results in rapid axonal retraction and NMJ destabilization, both structurally and functionally [8]. PSCs play a crucial role in muscle remodeling, with increased integration at the NMJ during development [9] and their intimate contact with perisynaptic satellite cells [10]. However, the morphology, ultrastructure and physiology of the NMJ can also display less substantial alterations in synaptic organization during a number of other stressors, such as exercise [1,11], inactivity/disuse [12,13,14], aging [15,16,17], crushing of the nerve/muscle [18,19], injury due to volumetric muscle loss (VML) [20] or the absence of associated proteins [21,22,23,24,25].

Functionally, NMJ remodeling ultimately affects neuromuscular transmission. Neuromuscular transmission is normally highly reliable, as each nerve impulse results in the release of more neurotransmitter (acetylcholine) than is required for evoking an action potential in the muscle fiber. This release of surplus transmitter and consequent excess depolarization of the post-synaptic membrane via AChRs is often referred to as the ‘safety factor’ [26], which ensures that a post-synaptic action potential will occur in response to each nerve impulse, at least in healthy tissue. A number of pathological conditions affecting the distribution of AChRs can lead to a reduction in the safety factor and impairment of neuromuscular transmission [26,27]. In an analogous manner, improper development, organization and remodeling at the NMJ can also impair the reliability and efficiency of neuromuscular transmission [28,29]. This review examines several such impacts on NMJ structure and function in the context of muscle disease, motor neuron disease, and aging.

## 2. The NMJ in Muscular Dystrophies

Muscular dystrophies are a group of degenerative skeletal muscle disorders, characterized by progressive skeletal muscle weakness, involving more than 40 genes and proteins in the extracellular matrix and basement membrane, sarcolemma-associated proteins, enzymes or proteins with putative enzymatic function or proteins in the nuclear envelope, sarcomere or endoplasmic reticulum [30]. Duchenne muscular dystrophy (DMD), the most common form of muscular dystrophy, is caused by the absence of the protein dystrophin, which plays a mechanical role in linking the contractile apparatus inside the muscle fiber to the extracellular matrix outside the muscle fiber. Dystrophin also binds directly or indirectly to a group of proteins at the sarcolemma collectively known as the dystrophin-associated protein complex (DAPC or DPC) or the dystrophin-glycoprotein complex (DGC). Dystrophin and the DGC play crucial roles in the regulation of various signaling pathways and accumulate at the NMJ post-synaptic membrane [31]. Dystrophin regulates synaptic homeostasis [32,33], endplate maintenance [25] and remodeling; however, it is not required for NMJ formation. EMG changes and abnormal NMJ morphology (increased fragmentation and discontinuity) have been identified in DMD patients [34,35,36,37]. 

The increased susceptibility to injury and muscle weakness is often attributed to the weakened muscle fiber cytoskeleton and abnormal intra-fiber signaling secondary to the loss of dystrophin and DGC organization. However, in the *mdx* mouse (the mouse model for DMD, which also lacks the protein dystrophin), muscle weakness has been linked to the NMJ [22,38,39,40,41], and a further loss in muscle function after injury is associated with additional changes in NMJ morphology (increased fragmentation and discontinuity). Such changes at the NMJ correspond to a reduction in synaptic transmission, such as decreased EMG amplitudes and increased neuromuscular transmission failure [42,43,44]. DMD patients also experience increased sensitivity and prolonged duration to neuromuscular blockage, further implicating NMJ abnormalities in the DMD pathophysiology [45,46]. Pre-synaptic structures in dystrophic muscles can also contribute to the disease phenotype, with increased discontinuity and branching of terminal nerves seen in *mdx* mice [44]. PSCs could also play a crucial role in driving disease pathology in *mdx* muscle, with a failure to cap the pre-synaptic nerve terminal, and abnormal organization of its extended processes [47].

Changes to the DGC, such as due to aberrant glycosylation of α-dystroglycan (associated with Fukuyama-type congenital muscular dystrophy), or absence of components of the DGC, such as α-dystrobrevin, dystroglycan (associated with dystroglycanopathy, limb girdle muscular dystrophy and congenital muscular dystrophy) [30] or syntrophin, results in altered NMJ morphology and function [21,31,48,49,50,51]. Animal models lacking some components of the DGC also display neuropathy [52]. However, absence of other DGC components, such as α- or γ-sarcoglycan (mutations of which are associated with limb-girdle muscular dystrophy) [53], does not appear to affect NMJ morphology [54,55]. Thus, alterations of the DGC alone do not predict NMJ morphology or function. It has been proposed that abnormal NMJ morphology results from cyclic muscle degeneration, local denervation, and reinnervation [56,57]; such hypotheses are compelling but remain to be thoroughly tested.

The activity of synaptic nuclei represents an alternative potential node for regulating NMJ structure and function. Separate from the many nuclei located along the myofiber (extra-synaptic nuclei), synaptic nuclei are a group of three to six functionally specialized nuclei that are anchored underneath the post-synaptic membrane of the NMJ and guide the expression of proteins supporting NMJ structure and function (reviewed in [58,59]). Key proteins in the nuclear envelope such as nesprin-1 (nuclear envelope spectrin repeat protein) and Sun-1 (Sad1 and UNC-84 domain containing protein) are involved in positioning and anchoring of the synaptic nuclei [60,61]. When these nuclear envelope proteins are experimentally deleted, the clustering of synaptic nuclei is altered (reduction in number of synaptic nuclei), but the NMJs appear normal [60]. On the other hand, concurrent with the neuromuscular remodeling and transmission deficits noted above, *mdx* mouse muscles display a marked increase in the number of synaptic nuclei [44]. Conversely, there is a reduction in the number of synaptic nuclei in patients with laminopathies (a class of dystrophies typically caused by mutations in the *LMNA* gene encoding Lamin A and C, which are nuclear scaffold proteins). These patients also display NMJ fragmentation and disorganization, often have abnormal EMG indicative of myopathy [62,63], and sometimes present axonal neuropathy [64]. In mouse models with *LMNA* mutations, NMJ morphology is similarly disorganized and fragmented, with mislocalized synaptic nuclei in addition to pathological axon histology [62,65]. 

The interactive roles of pre-synaptic and post-synaptic structures in driving dystrophic pathophysiology must be examined further. What is clear, though, is that although the NMJ may be unaffected in some dystrophies, it is clearly a significant disease modifier/driver in others.

## 3. The NMJ in Aging Muscle

Adult skeletal muscles decline in size with age [66], resulting in a loss of muscle mass (sarcopenia) and consequent weakness. The impact of muscle loss is exacerbated by the corresponding decline in the *quality* of the preserved muscle (e.g., amount of force per unit volume). These deficits, together with increased susceptibility to injury, reduced recovery, and proprioceptive decline [59,67,68,69], predispose the risk of falls and related injuries [70,71], which are linked to morbidity and mortality [72,73]. Sarcopenia has enormous social and economic benefits: a 10% reduction in prevalence alone would result in savings of well over a billion dollars [74]. Despite significant advances in understanding the molecular alterations in aging, the pathophysiology of age-associated muscle weakness remains unclear. 

Some describe sarcopenia as a primary muscular pathology, with only minimal changes in the peripheral nerves and motor units occurring much later than the onset of sarcopenia [75]. Indeed, aging muscles share several similarities to the muscle dystrophies described above. Synaptic nuclei in aged muscle have abnormal expression of nuclear proteins, such as reduced *LMNA* gene expression, suggesting that muscle dysfunction with aging may be similar to that seen in laminopathies [76]. Other similarities between aging muscle and dystrophic muscle include a loss of dystrophin with age. However, there is no consensus on other components of the DGC, with reports of increased, decreased and unchanged expression of DGC components [77,78]. 

However, the diminished muscle quality suggests additional neural contributions of to muscle wasting. A number of age-associated pathological changes have been reported in peripheral nerves and NMJs, which have even been posited to initiate and drive the muscle pathology in sarcopenia [79]. There are strong correlations between aging and deficits in axonal transport in peripheral neurons [16,19,80,81]. These deficits impair the delivery of vital synaptic and energetic cargoes to the pre-synaptic terminal and occur concurrent with age-associated changes in the neuronal cytoskeleton. Neurofilaments, the primary structural components of motor neurons and a key regulator of axonal caliber and cytoskeletal transport [82,83], appear particularly susceptible to age, based on observed changes in their density, organization, and phosphorylation state in aged mice [56,84,85].

Just as the NMJ dictates muscle physiology, it also influences muscle pathology. Several lines of evidence suggest that age-related changes in the NMJ play a key role in musculoskeletal impairment with aging [15,16,56,79,80,86,87,88]. Indeed there is increasing consensus that functional muscle denervation is a principal factor leading to sarcopenia [56,89], and some even describe sarcopenia primarily as a “disorder of the NMJ” [80]. Despite the continuing ambiguity of sarcopenia etiology, it is clear that, at a minimum, age-dependent changes in the peripheral nerve and NMJ contribute to the muscle pathology in sarcopenia [16,56,81,84]. 

Animal and human studies show that with aging, the pre-synaptic structures undergo degenerative changes typified by axonal denervation, reinnervation and remodeling [90,91,92] as well as altered intramuscular branching. NMJs in aged rodents are increasingly fragmented and lose their junctional folds [93,94]. They also contain a decrease in the number of pre-synaptic vesicles and number of nerve terminals [90,95] and dispersion in the area of motor endplates [95], as well as reductions in the number of AChRs [96] and binding affinity to the AChRs [97]. Although changes in the NMJ can depend on the muscle [95] and perhaps even its activity level [11,91,98], gradual deterioration with aging likely underlies changes in synaptic transmission, resulting in a functional denervation described for aged muscle. The preferential denervation of fast fibers with reinnervation via axonal sprouting from slow motor neurons results in a conversion from Type II (fast) fibers to Type I (slow) fibers [16]. Whether this motor unit remodeling is a cause or effect of sarcopenia is not clear.

PSCs may be additionally sensitive to age; with aging, concurrent to shallowing of junctional folds, PSCs have also been observed to retract [99] or aberrantly infiltrate into the synaptic cleft [88]. Both of these phenomena would serve to destabilize NMJ structure and transmission. The interplay among neurons, muscle fibers, and glial cells at the NMJ remains an important and growing focus of current and future research [87].

## 4. Neuromuscular Junction Disorders

Congenital myasthenic syndromes (CMS) and myasthenia gravis (MG) are inherited and autoimmune conditions, respectively, that affect the NMJ and result in weak and/or fatigable muscle weakness. There are many types of CMS caused by errors in different genes encoding NMJ proteins, only some of which are known. The onset and severity of CMS is variable. CMS muscle weakness typically begins in early childhood but can also appear in adolescence or adulthood; CMS can result in death in childhood due to respiratory difficulties or result in mild skeletal muscle weakness and fatigue [84]. Approximately 60% of patients with CMS have mutations in the genes encoding the AChR subunits, although patients with CMS have mutations in the genes encoding the pre-, intra- and post-synaptic components of the NMJ [84]. The functional impact of NMJ instability is a reduced muscle endplate potential, an increased threshold for post-synaptic activation and, thus, a decreased safety factor for neuromuscular transmission, despite normal sodium channel activity [100]. PSCs may also play a role in the pathology of these diseases, with invasion of synaptic space by PSC processes or absence of PSCs in patients with CMS [101,102].

Animal models of CMS include mice with the same specific NMJ gene mutations found in patients with CMS and with manipulations of genes encoding NMJ protein for replicating aspects of the disease phenotype (such as expression of AChR-γ in AChR-ε knockout mice to model AChR-deficiency) [103]. While mouse models allow for the examination of mechanisms underlying pathology and the testing of therapeutics, sometimes they do not accurately model the disease severity (i.e., in some CMS models the mice display a more severe, lethal phenotype than humans with the same mutations in Dok-7, loss of AChR-ε) [104,105].

Analogously, the most common type of myasthenia, MG, is an autoimmune neurological disorder characterized by defective transmission at the NMJ. The cause of MG is thought to be the breakdown of self-tolerance in the thymus [106,107]. The autoantibodies are produced by autoreactive B cells, but activation of CD4^+^ T cells is also required for the autoimmune process [108]. Thus, MG is a B cell- and T cell-dependent disease. The clinical presentation is heterogeneous, running the spectrum from affecting only muscles in certain groups, particularly those of the eyes (ocular myasthenia, the most common finding), to more generalized weakness involving multiple muscle groups (generalized MG). MG can result in significant morbidity, including respiratory weakness that requires ventilation, and even mortality (~5–9%), with more male patients than female [109]. MG is a rare disease, with incidence of approximately 3–9 cases per million. Onset of MG usually becomes apparent during adulthood, but symptom onset may occur at any age. 

A possible pathway targeted during the progression of MG is post-synaptic endplate stability, which is in large part due to controlled AChR clustering (Figure 1C), a well-studied phenomenon. Briefly, muscle-specific kinase (MuSK) is a transmembrane tyrosine kinase crucial for forming and maintaining the neuromuscular junction, and activation of the MuSK complex drives AChR clustering [110,111]. Agrin, which is secreted from the pre-synaptic terminal, interacts with low-density lipoprotein receptor-related protein 4 (Lrp4), which results in the repositioning of the Lrp4-MuSK complex, leading to the activation of MuSK through phosphorylation. Phosphorylated MuSK activates a downstream signaling pathway that leads to the focused clustering of AChRs. A collective attack on AChRs directly as well as their clustering has the potential to dramatically destabilize the NMJ. 

Consistent with such destabilization, most patients with MG raise autoantibodies against the acetylcholine receptors (AChRs) and sometimes to MuSK, Lrp4 and agrin [112,113]. AChR antibodies are found in most MG patients (~80%), predominantly of the IgG1 and IgG3 subclasses [114]. In addition to binding the AChRs, they activate the complement cascade, leading to the formation of the membrane attack complex, which causes damage of the post-synaptic membrane along the synaptic folds that contain AChRs and associated proteins, including voltage gated sodium channels [114]. Antibodies against MuSK are found in a small percent of MG patients (7–10%), with most being female [115]. Antibodies to MuSK interfere with the described role for MuSK in AChR clustering; however, they are of the IgG4 subclass and do not elicit a complement response or cell-mediated toxicity. There are some patients who do not have AChR or MuSK antibodies (double-seronegative MG) but likely have antibodies against yet unknown targets [116]. Lrp4 antibodies are present in some patients, often in females and with antibodies to agrin as well [112]. Structurally, changes to AChR density and localization result in reduced endplate size and complexity [100,117].

Animal models of MG produce key features of human disease, including antigenic modulation of the AChR, complement-mediated damage of the NMJ, and muscle weakness [108]. There are essentially two experimental models, either EAMG (experimental autoimmune MG) in which injected antigens elicit an ‘active’ immune response, or PTMG (passive transfer MG), in which injecting antibodies (either from a MG patient or EAMG animal) results in the ‘passive’ transfer of autoimmunity [108,118]. The antibodies for PTMG are administered by intravenous or intraperitoneal injection, especially into rats where disease symptoms are more distinguishable compared to mice [107,108]. Passive transfer has been often used to study the efficacy of new therapeutic interventions, given advantages such as ease of use (a single injection) and quick manifestation of symptoms (within days); however, the cellular mechanisms that drive antibody production are not present with PTMG. Although antibodies produce post-synaptic injury similar to the human disorder, there also can be extensive inflammation not seen in patients with MG [118]. Complement, part of the innate immune response, augments the adaptive immune response in MG, and there is evidence that it plays a central role in pathology. There is no consensus regarding which component(s) of the complement cascade is/are the optimal target; thus, targets have included a range of components in the complement cascade [118]. Despite their clear relevance to human pathophysiological progression and improvements in phenotype with some treatments, there is a dearth of information regarding NMJ morphology and function in animal models; such studies may allow us to better understand molecular mechanisms underlying neuromuscular failure in MG. 

Another disease of the NMJ includes Lambert-Eaton myasthenic syndrome (LEMS). LEMS is a rare neuromuscular immune disorder, with patients suffering from muscle weakness and autonomic dysfunction [119]. Patients with LEMS typically express antibodies against the pre-synaptic voltage gated calcium channels (VGCC), disrupting the ability of nerves to release acetylcholine. LEMS is paraneoplastic (i.e., an immune response to cancerous tumor) in more than half of the patients, with most patients associated with small cell lung carcinoma. Animal models include passive transfer of human VGCC antibodies into mice to elicit impaired neuromuscular transmission [120]. Other disorders of the NMJ also include illness from exogenous toxins that target the NMJ (such as botulism) and additional antibody-mediated disorders of the NMJ (such as neuromyotonia and Guillain Barre syndrome, among others) [121].

## 5. NMJ in Motor Neuron Diseases

Motor neuron diseases (MNDs) are a group of progressive neurological disorders that destroy motor neurons (reviewed in [122,123]). The pathogenesis of motor neuron diseases, which typically result in the loss/degeneration of upper or lower motor neurons, is still not fully elucidated. In this section, we will address the role of the NMJ in the two most common motor neuron disorders, amyotrophic lateral sclerosis (ALS) and spinal muscular atrophy (SMA). 

ALS is a progressive and fatal neurodegenerative disease affecting upper and lower motor neurons [124,125]. Patients with ALS have a broad clinical spectrum, with patients presenting either upper or lower motor neuron predominant symptoms. While a majority of ALS cases are sporadic, 5–10% of ALS cases are inherited (familial or fALS). The genetic defects that cause ALS are still being elucidated, although 50% of ALS cases result due to mutations in chromosome 9 open reading frame 72 (C9orf72), superoxide dismutase 1 (SOD1), transactive response DNA-binding protein (TARDBP) or fused in sarcoma (FUS) [126,127,128,129]. One hypothesis for ALS is that motor neuronal loss starts in a “dying-forward” process in the brain [130]. A contrary hypothesis is the “dying-back” process, in which the disease pathology begins at the NMJ [131]. In mouse models of ALS (SOD1^G93A^ mice, which overexpress human mutant SOD1, and TDP-43^Q331K^ mice, which express human mutant TDP-43), NMJ alterations occur prior to symptom onset [132,133]. In a FUS-ALS model, which is also characterized by NMJ defects, the enrichment of FUS protein in synaptic nuclei is disrupted [134]. Thus, irrespective of initiating factors, the NMJ dysfunction is a key early event in the pathogenesis of ALS. 

SMA is a neuromuscular gene caused by mutations in the survival motor neuron 1 gene (SMN1). The variability of age of onset and disease severity is due to the presence of its homolog, SMN2. Mouse models of SMA display NMJ abnormalities before a severe phenotype ensues [135]. The loss of SMN in mice with fully mature NMJ resulted in pathology only with injury and aging [136], while the loss of SMN before NMJ maturation resulted in a severe SMA-like phenotype. Thus, abnormal NMJ formation and maturation, due to the loss of SMN, play a crucial role in the pathogenesis of SMA [137]. 

**Figure 2:**
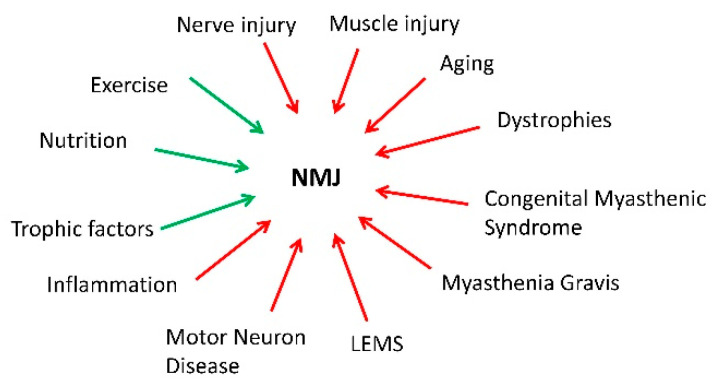
The neuromuscular junction (NMJ) as a central node in neuromuscular function. There are many conditions in which the NMJ plays a critical role or at least as a mediator of disease (red). However, some interventions can minimize NMJ dysfunction (green). LEMS: Lambert-Eaton Myasthenic Syndrome.

Overall, it is clear that the NMJ plays a significant role in a plethora of conditions that result in neuromuscular pathology (Figure 2). 

## 6. Therapeutic Approaches to NMJ Function

Gene therapy approaches are being investigated for ameliorating the pathophysiology of various muscular dystrophies. The hope is that gene and cell therapy strategies will continue to improve for treatment of DMD and other muscle diseases and will, as a consequence, also restore NMJ form and function [138]. For *mdx* mice, the correction of NMJ fragmentation requires a threshold of dystrophin restoration between 19% and 50% [40]. Restoration thresholds for other targets likely vary depending on their physiological roles. Inactivation of one target, MuSK, causes a reduction in AChR density and a change in the gross synaptic arborization of the endplate, which can lead to the complete loss of AChRs and disappearance of the synaptic structure [139]. In our previous work, we found a significant decrease in expression of MuSK in *mdx* mice [43], and others have shown that increasing expression of MuSK or rapsyn (a cytoplasmic MuSK effector protein) with adeno-associated viral vectors or transgenic overexpression of LRP-4 (which forms a complex with MuSK and binds to agrin) improves NMJ structure and protects *mdx* muscles from contraction-induced injury [38,140]. Gene therapy to increase expression of mini-agrins in mouse models of congenital muscular dystrophy improves survivability, motor and skeletal muscle function and skeletal muscle histopathology [141,142,143]. Utrophin upregulation through artificial transcription factors in *mdx* muscles results in an increase in the number of AChRs and reduced NMJ fragmentation [39]. This is accompanied by an improvement in muscle contractility, but it is difficult to tease apart the contribution of the NMJ versus other changes in the cell (e.g., improvement in sarcolemma stability, mechanotransduction of force, etc.).

NMJ fragmentation and denervation, and consequent decreased skeletal muscle contractility in aging skeletal muscle have also been mitigated by stabilizing endplates: for example, via transgenic expression of LRP-4 and α-sarcoglycan (which delays LRP-4 degradation). Gene therapy to increase expression of Dok-7 (a cytoplasmic adapter protein, which is a substrate of MuSK and activates MuSK kinase activity) [144] in aging skeletal muscle also improves the acetylcholine receptor area, innervation and consequent skeletal muscle contractility, further implicating the role of NMJ pathology in sarcopenia [145,146]. An engineered agrin (a C-terminal fragment of mouse agrin) improved skeletal muscle strength in a sarcopenia-like murine muscle model (neurotrypsin-overexpression) [147].

Interventions to stabilize the NMJ in mouse models of ALS and SMA (such as activation of MuSK using agonist antibodies and increasing agrin function using a synthetic agrin fragments) do not rescue the neuromuscular pathology but provide modest delays in NMJ decline [148,149]. Thus, therapeutic interventions to the NMJ in these motor neuron diseases could be a disease modifying therapy. Gene therapy approaches have been developed to ameliorate symptoms in patients with SMA. Gene therapy and other pharmacological therapies are currently being investigated for patients with ALS [123]. 

Caloric restriction (a consistent pattern of reducing average daily caloric intake) has been shown to extend life span and slow age-related chronic diseases in a variety of animal models, and there is growing enthusiasm about the potential for calorie restriction to possibly improve human longevity. The concept that a reduction in food intake retards the aging process and extends the life span of organisms of diverse phylogenetic groups is one of the leading paradigms in gerontology [150]. Caloric restriction lessens age-related declines in most physiological systems including the neuromuscular system [66,151,152,153]. This ability to delay the onset of age-related diseases appears to extend to age-related loss of motor neurons [154] and even changes in the NMJ [93]. Specifically, caloric restriction in mice up to 24 months of age shows that the NMJ is remarkably preserved, with damage to postsynaptic NMJ and axonal degeneration observed less frequently than in age-matched controls [93]. Fragmented and denervated post-synaptic sites were all significantly lower in these calorically restricted mice than in controls. Caloric restriction and the associated lowering of oxidative stress may help identify targets to preserve the NMJ with aging [16]. In addition, various diets can manipulate the activity of epigenetic modifying enzymes, allowing for changes in miRNA expression, DNA methylation and histone acetylation/deacetylation. Whether prolonged CR increases life span (or improves biomarkers of aging) in humans is unknown.

Pharmacological treatment strategies for MG include drugs for immunomodulation through immunosuppressants and steroids, thymectomy and drugs to improve neuromuscular transmission. Acetylcholinesterase inhibitors are also used to treat patients with MG, although this is not effective for all patients [155]. An engineered agrin, recalcitrant to anti-agrin antibodies, has been used to treat an animal model of MG, and the recombinant agrin fragment improves NMJ morphology (reduced NMJ fragmentation and nerve sprouting), transmission and increased MuSK in animal models of MG [113]. Complement inhibitors and targeted monoclonal antibody agents are being investigated in treating MG as well [155]. Treatment strategies for LEMS include drugs for immunomodulation, tumor resection and 3,4-diaminopyridine, which prolongs the duration of depolarization by blocking potassium ion efflux, improving the release of ACh vesicles [119,122]. 

Thus, there are numerous examples demonstrating the potential for targeting NMJ stability to improve muscle function or slow muscle decline. On the other hand, there is considerable diversity in whether a given molecular target directly impacts NMJ stability or may indirectly (and possibly less efficiently) stabilize NMJs by improving muscle structure and function or reducing muscle degeneration. 

## 7. Exercise

It is now clear that the NMJ in mature skeletal muscle is not a fixed permanent structure [3,4] but instead possesses a large degree of structural plasticity [1,5,56], remodeling in response to a variety of cues. This includes exercise, which can result in physiological adaptations (increased quantal content or neurotransmitter released), as well as morphological adaptations (larger number and length of terminal branches or changes in post-synaptic endplate size) that affect performance [86,156,157,158]. Resistance training can diminish functional loss in muscles despite only moderate increases in muscle mass, suggesting that the improvements are via neural adaptation [80,159]. Aging interferes with the ability of NMJs to adapt to exercise [86], but overall age-related changes in the NMJ are reduced with exercise [11,56,93,157,160].

The highly conserved Hippo pathway regulates several cellular processes. The main effectors of this pathway, Yap (Yes-Associated Protein) and Taz (Transcriptional co-activator with PDZ binding motif) are activated by exercise and interact with various signaling pathways to increase skeletal muscle size [161,162,163,164,165]. Muscles lacking Yap result in decreased muscle strength due to poor neuromuscular transmission, consistent with reduced AChR density and impaired clustering, reduced endplate occupancy, and reduced miniature endplate potential frequency [166]. Paradoxically, overexpression of Yap induces skeletal muscle atrophy [167], and increased Yap is seen in dystrophic and aged skeletal muscle [168,169,170,171]. Aging interferes with the ability of NMJs to adapt to exercise [86], and overall age-related changes in the NMJ are reduced with exercise. Yap activity in response to skeletal muscle loading is also altered in laminopathies and congenital dystrophies, with an absence of skeletal muscle hypertrophy with functional overload [172,173,174]. Thus, the blunted response to exercise in diseased or aging skeletal muscle [175] could be due to the Hippo pathway-mediated regulation of the NMJ. 

Due to the increased frailty of dystrophic muscles, exercise-mediated adaptations may be suppressed in patients with DMD compared to healthy subjects, and studies exploring the use of exercise as a therapeutic approach to improve skeletal muscle strength have been limited [176,177,178]. It is unclear whether exercise-mediated improvement in skeletal muscle strength and function [179] is due to neural adaptation or the NMJ, as is the case with improvements seen with an isometric exercise program in the *mdx* mouse [180]. Whole body low-intensity vibration improved skeletal muscle strength in a small study of DMD and BMD patients, presumably due to neural changes [181]. Exercise in *mdx* muscle increases utrophin expression [182], and increased utrophin expression in *mdx* muscle has been shown to decrease NMJ fragmentation and increase skeletal muscle strength [183,184]. Improvements in skeletal muscle following exercise were also seen in patients with FSHD, limb girdle muscular dystrophies, myotonic dystrophy and metabolic myopathies [185,186,187,188,189,190,191], although the role of neural adaptation remains unclear in these studies. For patients with MG, exercise has been shown to improve muscle strength, although it is not clear whether fatigability is improved, which could be due to the various exercise protocols used [192,193,194,195,196,197]. Caution must be used in appropriate use of exercise, with careful determination of intensity of exercise determined for each condition [185].

Exercise in healthy muscles can exert beneficial effects not only on muscle but also on NMJ morphology and function [56,86]. Endurance training affects the morphology of NMJs in young adults and has been studied as a measure to counter changes in the NMJ with aging [157]. Specific adaptations to exercise training include increases in the length and number of nerve terminal branches, a higher number of pre-synaptic vesicles, and an increased number and distribution of AChRs [1,56,86,198]. Exercise can induce activation of neurotrophic factors and other molecules, which also have a positive impact on NMJ morphology [199,200]. Furthermore, alterations of structure induced by endurance training are associated with significant NMJ functional changes, such as synaptic transmission. Resistance exercise appears to yield similar benefits for the NMJ, but to a lesser degree [156,201]. 

## 8. Conclusions

The focus on the NMJ as a contributor to weakness incurred with muscle injury, aging and muscle disease represents a shift from predominantly myo-centric views. While changes in both pre-synaptic motor neurons and the post-synaptic muscle fiber likely contribute to denervation in aging and muscle diseases, other cell types (e.g., Schwann cells, satellite cells, etc.) and other factors (trophic factors, mitochondrial function and oxidative stress) may also contribute to a functional decline of the neuromuscular system. The interplay between muscle health, nerve health and NMJ structure and function remain exciting areas of research in the context of aging and muscle disease.

## Data Availability

Not applicable.
